# Health beliefs about bottled water: a qualitative study

**DOI:** 10.1186/1471-2458-9-196

**Published:** 2009-06-19

**Authors:** Lorna A Ward, Owen L Cain, Ryan A Mullally, Kathryn S Holliday, Aaron GH Wernham, Paul D Baillie, Sheila M Greenfield

**Affiliations:** 1 Medical Student, University of Birmingham, Birmingham, B15 2TT, UK; 2 Primary Care Clinical Sciences, University of Birmingham Medical School, Birmingham, B15 2TT, UK

## Abstract

**Background:**

There has been a consistent rise in bottled water consumption over the last decade. Little is known about the health beliefs held by the general public about bottled water as this issue is not addressed by the existing quantitative literature. The purpose of this study was to improve understanding of the public's health beliefs concerning bottled mineral water, and the extent to which these beliefs and other views they hold, influence drinking habits.

**Methods:**

A qualitative study using semi-structured interviews, with 23 users of the Munrow Sports Centre on the University of Birmingham campus.

**Results:**

Health beliefs about bottled water could be classified as general or specific beliefs. Most participants believed that bottled water conferred general health benefits but were unsure as to the nature of these. In terms of specific health beliefs, the idea that the minerals in bottled water conferred a health benefit was the most commonly cited. There were concerns over links between the plastic bottle itself and cancer. Participants believed that bottled water has a detrimental effect on the environment. Convenience, cost and taste were influential factors when making decisions as to whether to buy bottled water; health beliefs were unimportant motivating factors.

**Conclusion:**

The majority of participants believed that bottled water has some health benefits. However, these beliefs played a minor role in determining bottled water consumption and are unlikely to be helpful in explaining recent trends in bottled water consumption if generalised to the UK population. The health beliefs elicited were supported by scientific evidence to varying extents. Most participants did not feel that bottled water conferred significant, if any, health benefits over tap water.

## Background

Demand for bottled water has consistently increased during the last decade, making bottled water the fastest growing segment of the non-alcoholic beverage market worldwide [1]. Consumption of bottled water in the UK rose from 1415 to 2275 million litres between 2000 and 2006 [2] and in 2003, UK consumers spent £1 billion on bottled water [3]. Indeed, for some of these consumers, bottled water has become a complete substitute for tap water [4].

However, these increases, in the UK at least, fly in the face of improving tap water quality over the last 10 years [5] and are even more surprising given that bottled water can cost on average 500–1000 times more per litre than tap water [6].

In contrast, recent media reports suggest a new-found scepticism about bottled water. A recent BBC Panorama documentary highlighted the environmental cost of bottled water [7]. There are also reports of the general public's reticence to accept the rising cost of bottled water on the basis of health claims [8,9].

### Literature review

No qualitative studies address public beliefs about bottled water in the English language peer-reviewed literature. A small number of quantitative studies have focused on this general area, although none have been carried out in the UK.

A discussion paper published in 2006 by Doria [1] reviewed the literature and identified dissatisfaction with tap water taste, odour and sight, and health concerns over tap water, as the major motivating factors for choosing bottled water. It also highlighted the lack of peer-reviewed literature on the reasons for choosing bottled water and the need for more research. In another discussion paper published on behalf of the Worldwide Fund for Nature (WWF) in 2001 [6], although there was some reliance on non-peer reviewed literature, similar suggestions were made indicating taste, safety and health issues to be the main motivating factors to buy bottled water.

The most recent original peer-reviewed research was an extensive quantitative study produced by the American Waterworks Association (AWWA) published in 2005 [10], a telephone survey of 2268 American residents. The findings of the study were that bottled water drinkers were satisfied with the quality and safety of tap water and suggested that most bottled water drinkers saw bottled water as a "luxury item", and not something that they purchased due to any perceived problems with their domestic water supply. It also identified taste, safety and healthiness as motivating factors in the choice to purchase bottled water as an alternative to tap water. However, whilst this study highlighted health beliefs as an important factor, it failed to explore what these consumers' health beliefs actually were.

A smaller telephone-interview-based quantitative study of residents of Quebec [11] concluded that taste, rather than safety, was the most important motivating factor for people buying bottled water. This study also noted consumer dissatisfaction with tap water. Research conducted in France in 1989, 1995 and 2000 [12] supports this, showing consistently that taste was a more frequently given reason for drinking bottled water than reasons concerning health and tap water risk.

In contrast, concerns over tap water safety and the consumption of bottled water as a substitute for other beverages were the motivating factors in over 80% of the 1600 Americans interviewed in an older study by the AWWA published in 1993 [13].

A large laboratory-based study by Olson [14] is often referenced in the literature. However, the claim made by the paper that "It is absolutely clear, therefore, that a leading reason for the explosion in bottled water sales is the public perception, fuelled by heavy industry advertising, that bottled water is pure and pristine, and thus a healthier choice than tap water" was not based upon any published research and was merely an expression of the author's opinion.

Existing quantitative literature broadly identifies health, taste, odour and sight as reasons for consumer preferences for bottled water. Health beliefs therefore seem to be an important factor in choosing to drink bottled water. However the existing research is not explicit about what these beliefs are and tends to take a comparative approach in identifying why the consumer might choose to drink bottled water as an alternative to tap water. Hence the health reasons given are generally beliefs concerning unfavorable properties of tap water. Previous research has been based in the USA, Canada and France and although these studies do seem to complement each other in the themes that arise, they concern different populations with different water supplies. It is unclear how generalisable the findings are to the UK.

This study was therefore designed to discover more about individuals' beliefs associated with bottled water and the perceived health effects of bottled water, in a UK setting. We also aimed to determine whether these beliefs were important factors in any motivation to drink bottled water and hence their possible contribution to the growth of the bottled water market.

## Methods

### Recruitment of participants

All participants in this study were users of the Munrow Sports Centre on the University of Birmingham campus. The majority of this sports centre's users are staff and students at the University of Birmingham, with a small number of members from the local community. This cohort was targeted based on the assumption that sports centre users might be more likely to have developed health beliefs than the general public. Recruitment occurred on six separate occasions at varying times of the day on different days of the week in January and February 2008 at the entrance to the sports centre. Every user entering or leaving the building during these times was approached.

Potential participants were briefed about the nature of the study by the researchers and after a provisional agreement to participate they were invited, by a standard e-mail on two occasions, to attend an individual interview at a time and place convenient to them. This ensured a relaxed atmosphere during the interview, with ample time in which to explore the interview themes. Each person who responded to the e-mail invitation was interviewed with no exclusions. Recruitment ended when all of the researchers agreed that no new themes were emerging from the content analysis and that data saturation had been reached.

### Data collection

Semi-structured interviews were deemed the most appropriate method of data collection for this topic for which there is a lack of previous research. Semi-structured interviews use some pre-determined, mainly open ended questions, to help define the area to be explored, but are flexible and allow the interview to diverge from this guide in order to pursue ideas in more detail [15].

A pilot study with four interviews was conducted, which informed the development of a basic interview schedule constructed from previous research findings in this area. The modified interview schedule was subsequently used for all of the interviews in this study. It prompted participants to discuss their habits of bottled water consumption, perceived differences between tap and bottled water, personal beliefs about bottled water and the beliefs that others hold about bottled water. It was emphasised that the term 'bottled water' referred to still bottled water purchased from a supermarket, shop or vending machine, and not sparkling water, or tap water in a bottle.

The interviews were conducted by six of the researchers (LW, OC, RM, AW, KH, PB) who were briefed in the techniques of qualitative interviewing by SG and practiced these techniques informally before data collection began. Two researchers were present at each interview, one conducting the interview and one operating the recording equipment. The interviews were recorded using digital recording equipment and later transcribed verbatim.

In total, 23 interviews were conducted between January and March 2008 in the participants' own homes, places of work, or at the University of Birmingham Medical School. The length of interviews ranged from 5 minutes 52 seconds to 22 minutes 12 seconds, mean length 12 minutes 17 seconds. The variation in length of the interviews was not related to the researcher conducting the interview. Instead the variability is mainly attributable to differences in participant knowledge and convictions about bottled water. The shortest interview resulted from the inclusion of one participant for whom English was not their first-language and hence they found it difficult to give extended answers to the questions.

### Data analysis

A qualitative content analysis using elements of grounded theory was employed to analyse the transcripts. The inductive process of identifying themes from the data collected, rather than applying top down *a priori *categories, was appropriate given the paucity of previous research exploring public perceptions of beliefs about bottled water [16].

The data was broken down into meaningful phrases or sentences using open coding. Categories were then identified and used to find the central themes that emerged from the data. In line with the grounded theory approach, these themes were not the same as those in the interview schedule but arose from the coding process. Investigator triangulation ensured trustworthiness of the analysis. The data was divided between the team with two coders for each transcript. Regular meetings of the team gave room for discussion about all levels of coding, particularly about data inconsistent with the evolving categories.

### Ethical considerations

This study did not require ethics committee approval. Participation was voluntary and participants were free to withdraw at any time. Participants were assured that confidentiality would be maintained. Before the interview began, the purpose of the study was explained again and informed verbal consent for participation in this study was obtained.

## Results

Socio-demographic information about each of the participants was collected at the beginning of each interview [see Additional file 1]. Of the 23 participants, there were 19 females and 4 males, aged between 18 and 52 years and all were currently living in the West Midlands Region. In terms of ethnicity the majority were White British, although those of White Irish, White Other and Asian British backgrounds were also represented. Eleven of the participants were students at the University of Birmingham, ten were employed by the University of Birmingham and two were employed outside of the University.

Participants were classified into three groups based on their reported average bottled water consumption in litres per week. The majority of participants in this study, 19 participants, were 'limited consumers' of bottled water and drank 0.5 to 3.5 litres per week. Two participants were 'consumers', drinking more than 10 litres each per week. Two participants were 'non-consumers' and never drank bottled water.

Data analysis revealed two main themes encompassing participants' beliefs about bottled water; health beliefs and environmental concerns. A third theme, factors that motivate participants to purchase bottled water also emerged [see Additional file 2]. Selected quotes are presented to illustrate the different subcategories that arose within these themes.

### Health beliefs about bottled water

Most participants believed that compared to tap water, bottled water conferred additional health benefits. Bottled water was considered to be a "healthy option" even if participants were unsure as to why:

*"Um...well I think it's probably better for you than er...it's probably got good minerals and stuff in it...um...probably better for you health-wise." *(P5, Limited consumer)

*"I mean I know it's good but I'm not sure why it's good." *(P11, Limited consumer)

Not only were the majority unsure as to why bottled water might confer health benefits, the nature of these health benefits was generally not specified either. In cases where participants did indentify a specific reason for the health benefit of bottled water compared with tap water, a belief that the minerals in bottled water conferred a health benefit was most commonly cited:

*"It has different minerals added, which I assume to have some benefit." *(P19, Limited consumer)

Only three participants gave any other specific health benefits. One participant believed that the bottled water could relieve symptoms of myalgic encephalopathy (M.E.), in the same way that filtering water seemed to provide some benefit to a family member:

*"Err...it started off with filter water. We used to buy filters and just filter all the tap water and it got too much hassle so we went onto bottled water and we went to filter water because my cousin got M.E. and they found that filtering his water pretty much cured all his symptoms of M.E. So when he...they started doing that we then started doing it as well and then." *(P6, Limited consumer)

A second participant believed that bottled water was "*better for babies*" (P15, Limited consumer), and the third person who ascribed a specific health benefit felt that bottled water could improve bone strength. However, as with many participants, they lacked conviction in their belief and were unable to explain their reasoning:

*"Um...er...it makes your bones stronger...I have no idea to be honest." *(P5, Limited consumer)

The majority of participants associated bottled water with having fewer impurities than tap water, and were more likely to trust the quality of bottled water than tap water:

*"They take out all the impurities don't they? And filter it all out." *(P16, Limited consumer)

*"It's a bit purer than what comes out of the tap." *(P14, Consumer)

Conversely, only two participants believed that tap water in the UK had either higher quality standards than bottled water or fewer impurities:

*"The quality control on tap water is much higher than on bottled water." *(P7, Limited consumer)

*"There is all sorts of erm...dubious erm...ingredients in the bottled water." *(P12, Limited consumer)

Despite their beliefs about the purity of bottled water, most participants expressed doubts as to the extent of the health benefits of bottled water compared with tap water:

*"I really don't think there is much difference between the two to be honest." *(P16, Limited consumer)

*"If the water quality of the water coming out of the tap is good I'm not sure there are any health claims that can be justified with bottled water"*. (P12, Limited consumer)

These views were not mutually exclusive with beliefs about health benefits, indeed most participants believed that bottled water did have health benefits, but that these benefits were negligible:

*"I suppose there must be some benefit I have no idea like you know what percentage of your recommended daily allowance it is...I certainly wouldn't drink it for minerals that otherwise I wouldn't get." *(P8, Limited consumer)

A minority of participants consistently denied any health benefits:

*"I don't think there is anything inherently better about bottled water than tap water." *(P1, Limited consumer)

A minority of participants expressed concerns that drinking bottled water was detrimental to health. One subject believed that the "*actual process of putting it into the plastic bottle*" (P21, Limited consumer) might impair the purity of the water and have a negative impact on consumers' health. Several other participants suggested a link between the plastic used for packaging and cancer. However, these concerns were about the repeated refilling of empty bottles with tap water rather than being about bottled water *per se*:

*"I have heard concerns about plastics and the chemicals from plastics getting into water and links to cancer." *(P19, Limited consumer)

*"What I've heard it did say something about the plastic starting to break down and something about it being able to cause cancer and as soon as anyone says something about cancer you'd think." *(P11, Limited consumer)

*"Um...it was about like the whole cancer of using the same bottle over and over again, it breaks down or something like that." *(P22, Limited consumer)

### Environmental concerns

A number of participants believed that it was *"not eco-friendly" *(P18, Non-consumer) to use bottled water and expressed concerns about bottled water's "carbon footprint". Some were concerned with the environmental impact of the plastic bottles:

*"I prefer to drink tap water because er it's got no packaging." *(P15, Limited consumer)

For others, the concern was with the environmental impacts of transporting the water:

*"I try to be green (laughs) and buy local mineral water...I try to buy something from the UK so it's not flown over." *(P4, Limited consumer)

When asked the reasons that may deter others from drinking bottled water, one person highlighted how topical the environmental issues were, by noting the impact of a recent BBC Panorama documentary:

*"Because they'll have watched programmes like the Panorama programme where they see the actual result of all this bottled water coming from all these countries where people can't actually get clean drinking water because we're stealing it all to have in our fancy bottles um and I dunno possibly because like we are here our we have filtered mineral water we don't have bottled water for staff*." (P17, Non-consumer)

### Motivating factors

Beliefs about the health benefits of bottled water emerged as a motivating factor influencing participants' decisions to drink bottled water in only a minority of cases, participants 5 and 11, both limited consumers:

*"Obviously mineral water has the extra natural goodness in it whereas tap water they probably add things to it to make it cleaned up." *(P11, Limited consumer)

Interviewer -*"Ok, and if we gave you a glass of tap water and a glass of mineral water and asked you to drink only one of them, which would you drink and why?"*

Participant- *"Um, the mineral water because it's gonna have more minerals in, it's gonna be fresher." *(P5, Limited consumer)

Others chose to drink bottled water because of concerns over the safety of tap water:

*"I'd probably choose the mineral water...because I think I have more confidence that it's been purified than the tap water." *(P19, Limited consumer)

However, most people were of the opinion that the tap water was fit for purpose and nobody expressed the view that they had reservations about the safety of tap water that were strong enough to prevent them from using it:

*"Tap water in England is fine, perfect" *(P9, Limited consumer)

Analysis revealed a number of other motivating factors that were unrelated to health beliefs. Convenience, taste and cost were almost universally important. The most commonly cited reason for purchasing bottled water was convenience:

*"When I go away to races I might not have a bottle with me so I'd buy some water..." *(P3, Limited consumer)

*"The only time I drink bottled water would be if I was out somewhere and wanted some water" *(P7, Limited consumer)

Several participants described how they would buy bottled water as they preferred the "taste" of it to tap water:

*"Well for me mineral water tastes of nothing...ha...cold nothing, I think tap water has some kind of taste to it..." *(P4, Limited consumer)

Participant 16 summarised the general consensus regarding the cost of bottled water:

*"If it was a choice to buy [bottled water] and not to buy it and it was drinkable from the tap then [they] would go for the tap water...it is mainly down to money." *(P16, Limited consumer)

Other factors included preference over other soft drinks, influence of the media, influence of marketing and advertising, bottled water as a status symbol or as a luxury item and re-use of the bottle as a container for tap water.

## Discussion

This study found that most people did hold health beliefs about bottled water, but that in the majority of cases these health beliefs were not strong motivating factors for purchasing bottled water. Other factors such as convenience, cost and taste emerged as far more important reasons for any preference for bottled water. In addition, most participants felt that there was not a significant health benefit in drinking bottled water compared to tap water. From this, it is unlikely that the recent surge in bottled water consumption is due to beliefs about health benefits associated with bottled water.

These results are important because until now, no qualitative studies have been conducted exploring public perceptions about bottled water and the factors that motivate people to buy it. The findings complement previous quantitative studies that have been conducted in this area [10-13]. The qualitative approach of this study allowed for a deeper exploration of the themes that were used in the quantitative data, and also gave room for new themes, not covered in the top-down approach of the quantitative studies, to emerge.

Convenience was a major motivating factor for buying bottled water, and one that has not been covered in previous quantitative studies. This may be because the 'top-down' approach of questionnaire design did not include convenience as a category. It seems obvious that people who would normally drink tap water would be motivated to buy bottled water when tap water is unavailable, for example in a shopping centre, or at the cinema. 'Convenience' is a motivating factor determined by the consumer's situation, not by the consumer's beliefs about bottled water.

Participants expressed health beliefs about bottled water that could be categorised as general health benefits or more specific health benefits. Although this is the first study to identify health beliefs about bottled water, the 2006 review by Doria suggests that there is much interest in the subject in both the grey literature and in the peer-reviewed literature, where unsupported claims regarding consumer beliefs are easy to find [1]. For example, Petrie and Wessely claim that bottled water is seen as a "natural antidote" to all the things bad for their health due to modernity [17].

A major emergent health belief was that most people were satisfied with the quality of their tap water supply and that it would not pose an adverse risk to their health. This is consistent with the data from Mackey *et al *[10], which demonstrated high tap water satisfaction, even in groups who drank bottled water in preference to tap water.

Interestingly, whilst the majority of participants expressed the belief that bottled water has health benefits of some kind, paradoxically these same participants also stated that the health benefits of bottled water are negligible or non-existent. This perhaps reflects confusion in the general public, as suggested by Olson [14], in that they only half-believe the marketing promoting health benefits of bottled water.

Such marketing might also explain why many participants, whilst able to state health beliefs regarding bottled water, were unable to explain or qualify these. The ability of marketing companies to create demand for bottled water "through the skilful use of language and image" has been discussed in a review of American culture [18]. This review suggests that in the public mind, purity, naturalness and healthiness are associated with bottled water through the specific marketing strategies of bottled water companies. The following statements taken from the websites of two leading brands seem to support this suggestion:

*"You want the best for your body and we've got it. Taste and feel the volvic difference, pure and natural...courtesy of Mother Earth" *[19]

*"Replenish your body with the purity of Evian" *[20]

There was some discrepancy between the specific health benefits participants believed bottled water to have and reality. Participants often felt that bottled water had an increased mineral content compared to tap water and that this conferred a health benefit. An extensive study conducted in the USA by Azoulay *et al *[21] compared the mineral content of tap water in various areas and a number of commercially available American and European bottled waters. Some brands of mineral water do indeed have a higher mineral content than tap water, which was found to be generally low in minerals, and were recommended as important dietary sources of calcium and magnesium. However, there is a considerable difference between bottled water brands, which no participant in our study seemed to be aware of.

Furthermore, in the USA study, some mineral waters were actually found to have a lower mineral content than the tap water supply, so the belief that all bottled waters are superior to tap water in terms of mineral content is incorrect. Although the study in question was conducted in the USA, the situation is likely to be similar in the UK. Moreover, whilst this study identified that some bottled waters provide a significant amount of the recommended daily intake for magnesium and calcium, none of these brands matched those that our participants drank. Of these, the preferred brands expressed all fell into the low mineral content classification of bottled water except for one, which was classified as moderate mineral content [21,22].

It is also important to remember that these minerals can be obtained from other sources in the diet, so the health benefits of the minerals contained in bottled water are not exclusive to this source. In addition, research shows that drinking waters with low mineral content does not lead to mineral deficiencies [23].

Where participants were able to give specific health benefits of bottled water we were not able to find supportive evidence in all cases. One participant believed that the symptoms of M.E were ameliorated by bottled water, which is something that existing literature does not appear to support. However, another participant mentioned that they felt that bottled water was especially benficial for babies. Despite finding no recommendations for this practice, we were able to find one study which suggested that choosing a mineral water with low sodium content may be useful in preparing formula milk because a hyperosmolar diet has been linked to hypertension and obesity in later life [23]. Having said this, no evidence could be found to suggest that tap water was unsuitable for this purpose.

Safety has previously been identified as an important motivating factor for buying bottled water [10-13]. Indeed, this was a theme that emerged in this study. It is worth emphasising again that participants did not feel that UK tap water was unsafe. Indeed the quality of this has continued to increase over the last 10 years [5], but participants still felt that bottled water was safer and purer when compared to tap water.

Only one participant correctly stated that tap water was in fact subject to more stringent testing than bottled water in the European Union (EU). The 1980 European Directive on natural mineral water outlines standards for these waters [24]. This became UK law in 1985 [25]. Under these regulations natural mineral water cannot be sterilised or otherwise treated to destroy microorganisms. Bottled water is not free of microorganisms as some might believe and this has been demonstrated by numerous studies [4,26-28].

Although European regulations are considered more rigorous than those in the USA [26], natural mineral waters are only tested every two months by independent laboratories, compared to tap water which is tested every two days in urban areas [5]. In addition, quality controls for tap waters are based on 62 parameters, compared to only 26 for mineral waters [5].

Doria notes that whilst there have been outbreaks of disease attributable to tap water, such as in Sydney in 1998, which led to an increase in bottled water sales, bottled water is not without similar events. The well-known brand Perrier was contaminated with benzene in 1990 and in 2004, Coca-Cola withdrew Dasani, its own bottled water, due to concerns about the levels of a potential carcinogen in the water [1,29].

A number of participants expressed concerns about a link between the plastic container of bottled water and cancer. A carcinogenic substance known as DEHA (di-ethylhexl adipate) is indeed used in the manufacture of PET (polyethylene terephthalate), a plastic used to manufacture most bottled water containers [27]. However, laboratory studies performed by the US Environmental Protection Agency concluded that leaching of DEHA from the bottle is not harmful to human health [30]. Although not true, the concerns held by participants about the plastic bottles are not irrational and replicate concerns that other people seem to have. For example, in 2004 a hoax e-mail circulated in the USA, attributed to Johns Hopkins University, suggesting that the plastic used to manufacture the containers for bottled water contained harmful dioxins, which is untrue [31].

Almost a third of participants expressed concerns over the environmental impact of bottled water. These concerns mirror recent media interest in the subject [7-9]. These concerns included comments about the 'carbon footprint' created by the transport of imported bottled water. A 2006 Earth Policy Institute study found that the British bottled water industry generates about 30,000 tonnes of carbon dioxide per year, which was estimated to equal the energy consumption of 6,000 homes per year [32].

The environmental impact of the plastic bottles themselves in their production and disposal was also mentioned by some participants. Packaging is generally made from plastics, either polyvinylchloride (PVC) or PET; the latter is becoming widely used as it is easier to recycle than PVC and does not release chlorine when burnt [5]. In the USA, annual production of PET to meet the needs of the bottled water industry uses around 18 million barrels of oil [32], which is a finite resource. Although the smaller UK market would mean lower oil use, considering that in 2006/2007, only 58.4% of municipal and 28.6% of household waste was recycled in the West Midlands, participants are probably correct in their concerns [33].

### Limitations

This study has several limitations. Selection bias may have occurred in that the participants in the study had both the time available and the inclination to take part. This might mean that those with particularly strong views on the issue were more likely to volunteer, but this does not appear to have been borne out in our results. Availability bias may have occurred in that the issues surrounding bottled water can quite feasibly change over time and certain factors may become transiently important [34]. An example would be negative health beliefs about tap water as a motivating factor to purchase bottled water following media reports of contamination of the supply.

The fact that all of the participants in this study had connections with the Munrow sports centre, and the majority were employed by or studying at the University of Birmingham, has implications for the generalisability of the findings of this study. Hence, the results may not be applicable to people who are unemployed or not in full time education, or people who are not sports centre users. Repetition of the study with a sample more representative of the general population may therefore be of value.

Respondent validation may have proved useful since respondents' reactions to emerging findings can help to refine explanations and can strengthen the rigor of thorough qualitative research [35].

### Recommendations

A number of issues arise from this study which may warrant further research. Namely the link between marketing strategies for bottled water and their role in creating health beliefs in the general public. It would also be interesting to see if it is possible to identify people who drink exclusively bottled water and question them about their reasons for this and their health beliefs about bottled water. Such information could then be compared with the results of this study to determine whether people who only drink bottled water are motivated to buy it by the same factors as the participants in this study, and the role of health beliefs within this. Finally, given the lack of knowledge about the purification process and safety of tap water in the UK found by this study, it could be useful to further educate the public with regard to the safety of tap water considering the prevalence of concerns arising that appear to be unfounded.

## Conclusion

The participants of this study had a range of health beliefs about bottled water which could be classified as general and specific benefits. Participants also held a number of beliefs about the impact the bottled water industry has on the environment. Although the majority of participants believed that bottled water had some health benefits, these beliefs played a minor role in determining bottled water consumption and are unlikely to be helpful in explaining the recent trends in bottled water consumption. The health beliefs that participants held were supported by scientific evidence to varying extents.

Convenience, cost and taste were more influential factors for participants when deciding whether to buy a bottle of water or not. Most participants did not feel that bottled water conferred significant, if any, health benefits over tap water.

## Competing interests

The authors declare that they have no competing interests.

## Authors' contributions

SG conceived of the study, provided support and advice to the other authors at all stages and was involved with redrafting of the manuscript. LW, OC, RM, KH, AW, PB were involved with the study design, recruitment, data collection, data analysis and writing of the manuscript. All authors read and approved the final manuscript.

## Pre-publication history

The pre-publication history for this paper can be accessed here:



## Supplementary Material

Additional file 1 **Socio-demographic backgrounds of the participants**. A table presenting the socio-demographic backgrounds of the participants. Click here for file

Additional file 2 **Themes identified in analysis and participants who contributed to these themes**. A table presenting the themes identified in the analysis and participants who contribute to these themes. Click here for file
