# Supplementary material for: Health beliefs about bottled water: a qualitative study
Source: BMC Public Health. 2009 Jun 19;9:196. doi: 10.1186/1471-2458-9-196 (PMC2714301; doi:10.1186/1471-2458-9-196)
Supplement: Additional file 1 — Socio-demographic backgrounds of the participants. A table presenting the socio-demographic backgrounds of the participants. [file 1471-2458-9-196-S1.doc]

| **Participant** | **Sex** | **Age group (Years)** | **Ethnicity** | **Occupation** | **Current Smoker**  **(Yes/ No)** | **Alcohol consumption**  **(Units per week)** | **Exercise**  **(Hours per week)** | **Bottled water consumption** |
| --- | --- | --- | --- | --- | --- | --- | --- | --- |
| **P1** | Male | 20-24 | White British | University student | No | 0-4 | 5-9 | Limited consumer |
| **P2** | Female | 15-19 | White Irish | University student | No | 0-4 | 10-14 | Limited consumer |
| **P3** | Female | 25-29 | White Irish | University employee | No | 0-4 | 10-14 | Limited consumer |
| **P4** | Female | 25-29 | White Other | University employee | No | 10-14 | 0-4 | Limited consumer |
| **P5** | Female | 20-24 | White British | University student | No | 10-14 | 10-14 | Limited consumer |
| **P6** | Female | 20-24 | White British | University student | No | 0-4 | 0-4 | Limited consumer |
| **P7** | Female | 20-24 | White British | University student | No | 5-9 | 0-4 | Limited consumer |
| **P8** | Male | 35-39 | White British | Non-university employed | No | 20-24 | 0-4 | Limited consumer |
| **P9** | Female | 20-24 | White British | University student | No | 0-4 | 10-14 | Limited consumer |
| **P10** | Female | 15-19 | White British | University student | No | 5-9 | 0-4 | Limited consumer |
| **P11** | Female | 15-19 | White British | University student | No | 10-14 | 0-4 | Limited consumer |
| **P12** | Male | 50-54 | White Other | University employee | Yes | 25-29 | 0-4 | Limited consumer |
| **P13** | Male | 25-29 | Asian British | University employee | No | 0-4 | 0-4 | Limited consumer |
| **P14** | Female | 20-24 | White British | University employee | No | 5-9 | 0-4 | Consumer |
| **P15** | Female | 20-24 | White Other | University student | No | 0-4 | 4-9 | Limited consumer |
| **P16** | Female | 40-44 | White British | Non-university employed | No | 0-4 | 0-4 | Limited consumer |
| **P17** | Female | 25-29 | White Irish | University employee | No | 0-4 | 0-4 | Non-consumer |
| **P18** | Female | 45-49 | White British | University employee | Yes | 20-24 | 0-4 | Non-consumer |
| **P19** | Female | 40-44 | White British | University employee | No | 5-9 | 0-4 | Limited consumer |
| **P20** | Female | 40-44 | White British | University employee | No | 5-9 | 0-4 | Consumer |
| **P21** | Female | 20-24 | White British | University employee | No | 0-4 | 0-4 | Limited consumer |
| **P22** | Female | 20-24 | Asian British | University student | No | 0-4 | 0-4 | Limited consumer |
| **P23** | Female | 15-19 | White British | University student | No | 0-4 | 0-4 | Limited consumer |

**Socio-demographic backgrounds of the participants**
