# Supplementary material for: Health beliefs about bottled water: a qualitative study
Source: BMC Public Health. 2009 Jun 19;9:196. doi: 10.1186/1471-2458-9-196 (PMC2714301; doi:10.1186/1471-2458-9-196)
Supplement: Additional file 2 — Themes identified in analysis and participants who contributed to these themes. A table presenting the themes identified in the analysis and participants who contribute to these themes. [file 1471-2458-9-196-S2.doc]

**Themes identified in analysis and participants who contributed to these themes**

|  | **P1** | **P2** | **P3** | **P4** | **P5** | **P6** | **P7** | **P8** | **P9** | **P10** | **P11** | **P12** | **P13** | **P14** | **P15** | **P16** | **P17** | **P18** | **P19** | **P20** | **P21** | **P22** | **P23** |
| --- | --- | --- | --- | --- | --- | --- | --- | --- | --- | --- | --- | --- | --- | --- | --- | --- | --- | --- | --- | --- | --- | --- | --- |
| **Health beliefs about bottled water** |  |  |  |  |  |  |  |  |  |  |  |  |  |  |  |  |  |  |  |  |  |  |  |
| General health benefits |  |  | x |  | x | x |  | x |  |  |  | x |  | x | x |  |  |  | x |  |  |  | x |
| Specific health benefits |  |  |  |  | x | x |  |  |  |  |  |  |  |  | x |  |  |  |  |  |  |  |  |
| Bottled water is purer than tap water |  |  |  | x |  |  | x | x | x | x | x | x | x | x | x | x | x |  | x | x | x | x |  |
| Health benefits of bottled water are  negligible |  | x | x | x |  | x |  |  |  | x |  | x | x | x | x | x | x |  |  |  | x |  | x |
| Definitely no health benefits to bottled  water | x |  |  |  |  |  | x |  |  |  |  |  |  |  |  |  |  | x |  | x | x | x |  |
|  |  |  |  |  |  |  |  |  |  |  |  |  |  |  |  |  |  |  |  |  |  |  |  |
| **Environmental concerns** |  |  |  | x |  |  | x |  |  |  |  |  |  |  | x |  | x | x |  | x |  |  | x |
|  |  |  |  |  |  |  |  |  |  |  |  |  |  |  |  |  |  |  |  |  |  |  |  |
| **Motivating factors to buy bottled water** |  |  |  |  |  |  |  |  |  |  |  |  |  |  |  |  |  |  |  |  |  |  |  |
| Health benefits* |  |  |  |  |  |  |  |  |  |  | x |  |  |  |  |  |  |  |  | x |  |  |  |
| Concerns about tap water* |  |  | x |  |  |  |  |  |  | x |  |  | x | x | x | x |  |  |  |  |  |  |  |
| Convenience | x | x | x | x |  | x | x |  |  | x | x | x | x | x |  |  | x |  | x |  | x | x | x |
| Taste | x | x | x | x |  |  | x | x | x | x |  | x | x |  | x |  | x |  | x | x |  |  |  |
| Preference over other soft drinks |  |  |  |  |  |  |  |  |  |  |  |  | x | x | x | x | x |  |  |  |  |  |  |
| For the bottle |  |  |  |  |  |  |  |  |  |  | x |  |  |  |  |  |  |  |  |  |  | x |  |
| Status symbol |  |  |  | x |  |  |  |  |  |  |  | x |  |  |  | x |  |  |  |  |  |  |  |
| Luxury item |  |  |  |  |  |  |  | x |  |  |  |  |  |  |  |  |  |  |  |  |  |  |  |
| Media |  |  |  | x |  |  |  |  |  |  |  |  |  | x |  |  |  |  |  |  |  |  |  |
| Marketing and advertising |  |  |  |  | x |  |  |  |  | x |  | x |  | x |  |  |  |  |  |  |  |  |  |

*Health beliefs as motivating factors to buy bottled water
